# Age-Dependent Changes in Effective Dose in Pediatric Brain CT: Comparisons of Estimation Methods

**DOI:** 10.3390/tomography10010002

**Published:** 2023-12-24

**Authors:** Yusuke Inoue, Masahiro Mori, Hiroyasu Itoh, Kohei Mitsui, Hiroki Miyatake, Takuro Yamane, Hirofumi Hata

**Affiliations:** 1 Department of Diagnostic Radiology, Kitasato University School of Medicine, Sagamihara 252-0374, Japan; wtp2016@gmail.com (M.M.); km19901004@gmail.com (K.M.); urokutanemaya@yahoo.co.jp (T.Y.); 2 Department of Radiology, Kitasato University Hospital, Sagamihara 252-0375, Japan; hiroyasu@kitasato-u.ac.jp (H.I.); hm99m2002@yahoo.co.jp (H.M.); kmri@kitasato-u.ac.jp (H.H.)

**Keywords:** age, brain, computed tomography, effective dose, pediatrics, radiation dose

## Abstract

The effective dose (ED) in computed tomography (CT) may be calculated by multiplying the dose–length product (DLP) by a conversion factor. As children grow, automatic exposure control increases the DLP, while the conversion factor decreases; these two changes affect the ED in opposite ways. The aim of this study was to investigate the methods of ED estimation according to age in pediatric brain CT. We retrospectively analyzed 980 brain CT scans performed for various clinical indications in children. The conversion factor at each age, in integer years, was determined based on the values at 0, 1, 5, and 10 years provided by the International Commission on Radiological Protection (ICRP), using a curve (curve method) or lines (linear method). In the simple method, the ED was estimated using the ICRP conversion factor for the closest age. We also analyzed the ED estimated by a radiation dose management system. Although the median DLP at each age increased with age, the median ED estimated by the curve method was highest at 0 years, decreased with age, and then plateaued at 9 years. The linear method yielded mildly different results, especially at 2 and 3 years. The ED estimated by the simple method or the radiation dose management system showed inconsistent, up-and-down changes with age. In conclusion, the ED in pediatric brain CT decreases with age despite increased DLP. Determination of the conversion factor at each age using a curve is expected to contribute to estimating the ED in pediatric CT according to age.

## 1. Introduction

Computed tomography (CT) delivers relatively high radiation doses, and the potentially detrimental effects of CT radiation exposure are a significant concern in modern medicine [1]. Evaluation of brain abnormalities is the most common indication of CT in children [2,3,4]. In the dose survey for pediatric CT in the USA, 52.6% of the analyzed examinations were brain CT excluding the scans of the sinus and maxillofacial area (4.3%, 2.8%, 7.0%, and 38.5% for 0, 1, 2–5, and 6–18 years, respectively) [3]. Children are radiosensitive, and their long life expectancies allow cancer to develop after a long latency period [5]. Epidemiological studies have demonstrated an increased incidence of brain tumors in children who have undergone brain CT [6,7,8,9]. Therefore, radiation dose management and optimization are essential for pediatric brain CT.

The volume CT dose index (CTDIvol) and dose–length product (DLP) are provided automatically by the CT scanner and commonly used as indices of the radiation dose in CT [10]. For brain CT, the CTDIvol is calculated from the absorbed dose measured using a 16 cm dosimetry phantom and the scanner parameters, and the DLP is an integral of the CTDIvol over the scan range. Use of the diagnostic reference level (DRL) is recommended to optimize the radiation dose in radiological imaging [11,12]. DRL quantities are defined for each imaging modality, and the CTDIvol and DLP are defined as DRL quantities in CT.

Because different DRL quantities are used for different imaging modalities [11,12], comparison of the radiation dose between different modalities is difficult. For radiation dose management and radiation risk communication, possible biological effects are concerned. The biological effect of a fixed absorbed dose varies among organs and tissues. The effective dose (ED) is estimated considering the type of radiation and the radiosensitivity of irradiated tissues and organs. It is accepted as an index of the risk of stochastic effects, including cancer induction, and facilitates the comparison of risks derived from different radiation sources. The International Commission on Radiological Protection (ICRP) offers conversion factors from the DLP to the ED, and the ED is often calculated as a product of the DLP and the conversion factor [10]. The conversion factor differs depending on the imaging region. Although the CTDIvol is generally higher for brain CT than for body CT, the conversion factor is much lower for brain CT, resulting in a lower ED. The conversion factor also varies according to age, and the ICRP offers values at 0, 1, 5, and 10 years in addition to adult values.

At a given level of X-ray output, fewer X-ray photons reach the detector in a larger patient, due to more severe attenuation. Stronger radiation exposure is required for a larger patient to preserve image quality. Automatic exposure control (AEC) assesses the degree of X-ray attenuation by the patient, primarily based on the localizer image, to define the scan range and automatically modulates the tube current and, consequently, radiation exposure [13,14]. Although approximately half of the facilities in previous surveys did not use AEC for pediatric brain CT [15,16], AEC achieves appropriate radiation dose modulation according to the head size in pediatric brain CT [17]. Therefore, applying AEC is recommended for optimization. 

The head grows rapidly early after birth, preceding the growth of the body, followed by a period of slower growth [18]. According to the head’s growth, the CTDIvol and DLP are higher in older children than in younger children [3,15,19,20,21]. However, the conversion factor from the DLP to the ED is higher in younger children [10]. The age-dependent changes in the DLP and conversion factor affect the ED in opposite ways. The ICRP offers the conversion factors at 0, 1, 5, and 10 years, and the method of conversion at other ages (for example, 2 years) is not defined explicitly. In this study, we analyzed pediatric brain CT where radiation exposure was modulated using AEC and evaluated the ED in relation to age using four practical methods. The aim of this study was to investigate the methods of ED estimation according to age.

## 2. Materials and Methods

### 2.1. Subjects

We retrospectively analyzed brain CT scans performed for various clinical indications in children aged <15 years at a single institution. The data were used in previous studies for different purposes [17,21,22,23]. The ED was not assessed in those studies. The Institutional Review Board approved this study, and the need for informed consent was waived due to its retrospective design. For patients who underwent CT examinations repeatedly, those performed at an interval longer than 1 year were included in the analysis. The exclusion criteria were as follows: lack of data on weight, weight > 80 kg, helical-mode imaging, erroneous use of an adult protocol, and no use of a head holder. Finally, 980 examinations (544 males and 436 females) were considered eligible.

### 2.2. Imaging Procedures

The imaging procedures were as described previously [21]. Briefly, two 64-detector-row CT scanners with the same specifications (Optima CT 660 Discovery Edition; GE Healthcare, Milwaukee, WI, USA) were used. After obtaining posteroanterior and lateral localizer images, axial CT images parallel to the orbitomeatal line were acquired in axial mode from the inferior margin of the posterior fossa to the top of the brain. The tube current was determined using AEC software (Auto mA and Smart mA; GE Healthcare), with a noise index of 4 [13]. Organ dose modulation was applied over the orbit to reduce the radiation dose to the eye lens [24,25]. The other imaging parameters were as follows: tube voltage, 120 kV; rotation time, 1 s; beam collimation, 0.625 mm × 16; slice thickness, 5 mm; slice increment, 5 mm.

### 2.3. Estimation of ED

The conversion factors from the DLP to the ED provided by the ICRP are 0.011, 0.0067, 0.0040, and 0.0032 mSv/mGy/cm at 0, 1, 5, and 10 years, respectively [10]. We estimated the ED by three methods based on the ICRP conversion factors: the curve method, linear method, and simple method. The conversion factor at each age in integer years (0, 1, 2, …, 14 years) was determined, and the scanner-derived DLP was multiplied by the conversion factor to estimate the ED.

In the curve method, the following equation was fitted by the nonlinear least squares method to the relationship between the conversion factor and age:

y = a(x + b)^c^

where x is age in years, y is the conversion factor, and a, b, and c are constants. R software (version 4.2.1; R Foundation for Statistical Computing, Vienna, Austria) was used for fitting. A conversion factor at a given age in integer years was calculated using the obtained equation.

In the linear method, the conversion factors at 2–4 and 6–9 years (integer years) were calculated by linear interpolation of those at 1 and 5 years and at 5 and 10 years, respectively. The conversion factors at 11–14 years were calculated by linear extrapolation of those at 5 and 10 years. In the simple method, the conversion factors at 0, 1, 5, and 10 years provided by the ICRP were applied to patients at 0, 1–2, 3–7, and 8–14 years (integer years), respectively. The relative conversion factor for the linear method at each age was calculated as the ratio of the conversion factor for the linear method to that for the curve method. The relative conversion factor for the simple method was calculated similarly.

In addition to the three conversion-factor-based methods, we analyzed EDs estimated automatically by the radiation dose management system Radimetrics (version 3.4.0; Bayer Medical Care Inc., Indianola, PA, USA) (Radimetrics method). Radimetrics uses Monte Carlo simulation for the estimation. The phantom used for the simulation is primarily selected based on age, and different phantoms are applied at <0.5, 0.5–2.5, 2.5–7.5, 7.5–12.5, and <12.5 years [26].

### 2.4. Data Analysis

The median values of the DLP and the EDs estimated by the curve, linear, simple, and Radimetrics methods were determined at each age in integer years. For example, the age of a child 2 years and 6 months after birth was regarded as 2 years. Additionally, age grouping was applied, and the median values of the DLP and the EDs estimated by the four methods were also determined for the following age groups: 0, 1–4, 5–9, and 10–14 years. Furthermore, the ED estimated by the curve method for each scan was plotted against actual age, considering years, months, and days of birth. For example, the age of a child 2 years and 6 months after birth was regarded as 2.5 years. Additionally, we calculated the ratio of the ED estimated by the Radimetrics method to the DLP, and we examined the relationship between the ratio and age in actual numbers.

## 3. Results

The following equation was successfully fitted to the relationship between age and the conversion factor:

y = 0.007392 (x − 0.3298)^−0.3586^.

The residual standard deviation was 0.00005.

**Figure 1:**
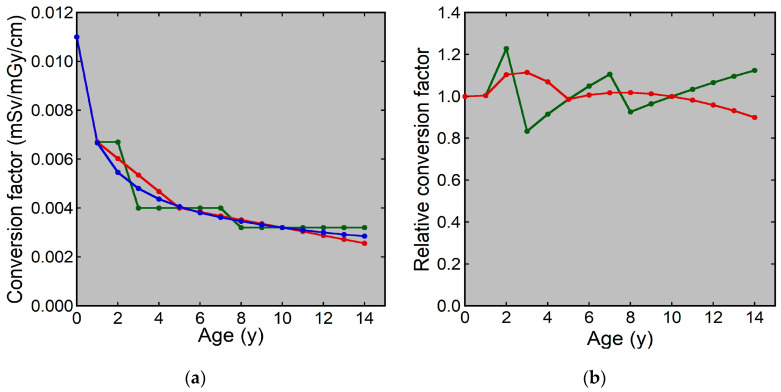
Conversion factors from the DLP to the ED at each age: (**a**) Conversion factors for the curve (blue), linear (red), and simple (green) methods. (**b**) The relative conversion factor for the linear (red) and simple (green) methods.

We calculated the conversion factor for the curve method using this equation at each age in integer years. The conversion factors for the curve, linear, and simple methods were almost identical at 0, 1, 5, and 10 years; however, there were discrepancies for the other ages (Figure 1). The conversion factor for the curve method decreased with increasing age, and the annual decrease became less evident gradually. The age-dependent changes appeared smooth. The conversion factors for the linear method were larger at 2, 3, and 4 years and smaller at 13 and 14 years than those for the curve method. The relative conversion factor for the linear method was 1.11 and 0.90 at 3 and 14 years, respectively. Discrepancies between the curve and simple methods were evident, especially at 2 and 3 years. The relative conversion factor for the simple method was 1.23 and 0.83 at 2 and 3 years, respectively.

**Figure 2:**
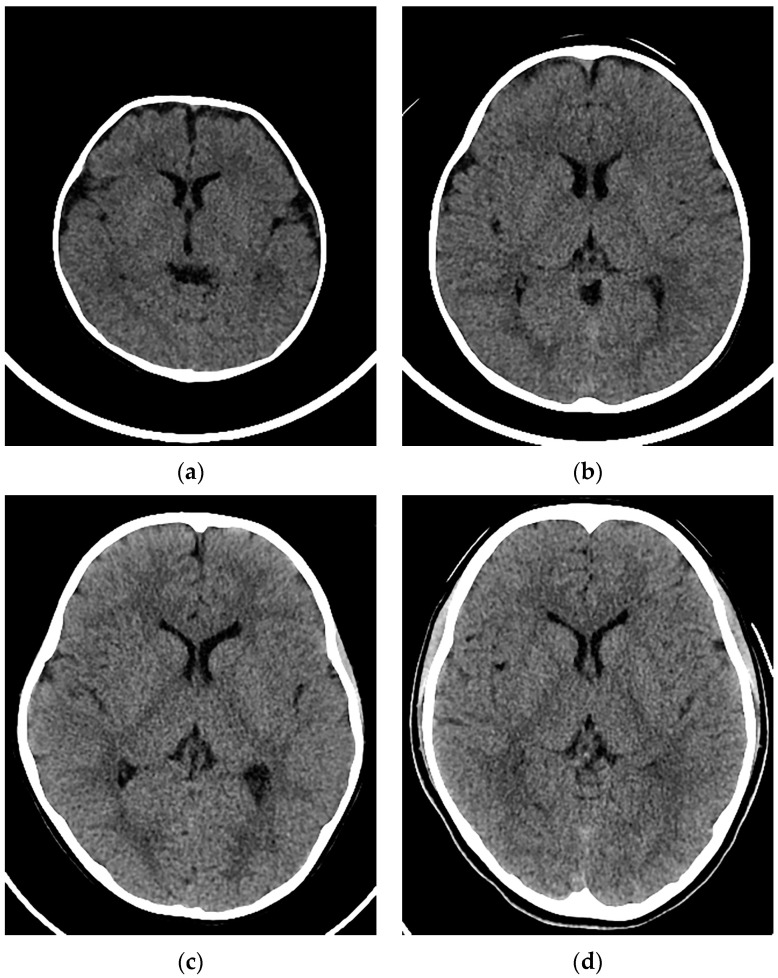
Examples of CT images in a (**a**) 3-month-old girl, (**b**) 1-year-old boy, (**c**) 5-year-old girl, and (**d**) 10-year-old girl.

**Figure 3:**
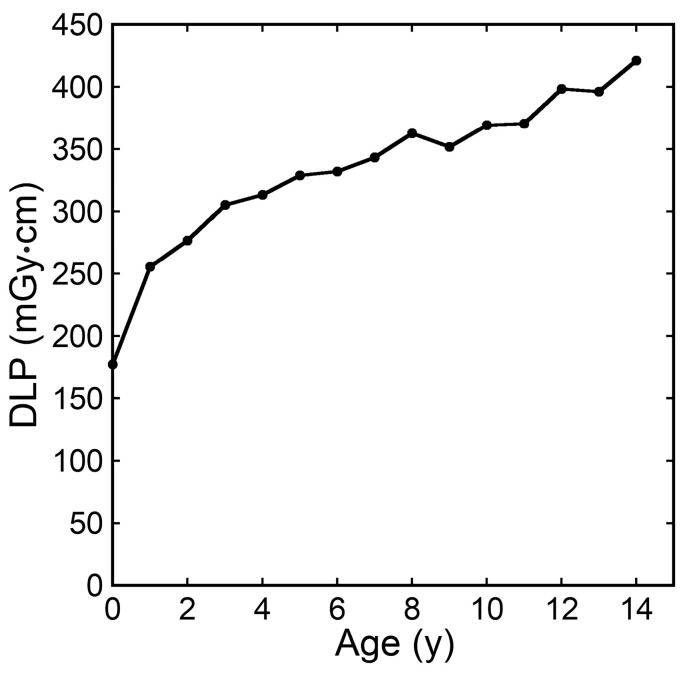
The median DLP at each age in integer years.

**Figure 4:**
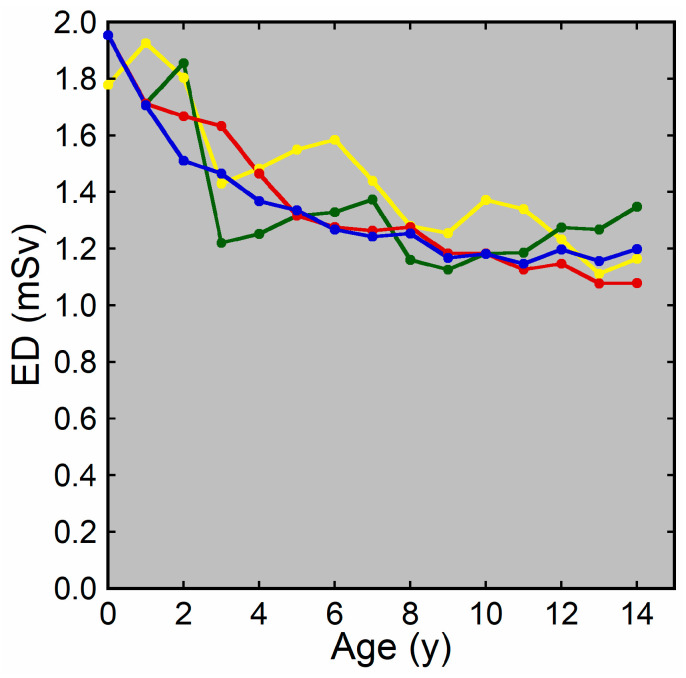
The median ED at each age in integer years. EDs were estimated by the curve method (blue), linear method (red), simple method (green), and Radimetrics method (yellow).

Examples of CT images at 0, 1, 5, and 10 years are presented in Figure 2. The median DLP at each age in integer years was lowest at 0 years and increased with age (Figure 3). The annual increase was relatively large between 0 and 1 years. In contrast, the median ED estimated by the curve method was highest at 0 years and decreased with age (Figure 4). The annual decrease was relatively large between 0 and 1 years and between 1 and 2 years. After that, the median ED decreased slowly and remained almost constant after 9 years. The median ED estimated by the linear method also decreased consistently with age. However, the ED–age profile appeared mildly deformed, creating a shoulder, at 2 and 3 years. The relationship between age and ED was complicated for the simple method. The median ED was high at 0, 1, and 2 years and decreased suddenly at 3 years. After that, it increased slowly up to 7 years, and then it decreased again at 8 years, followed by a slow increase. The median ED obtained by the Radimetrics method tended to decrease with age, but there were obvious up-and-down changes.

**Figure 5:**
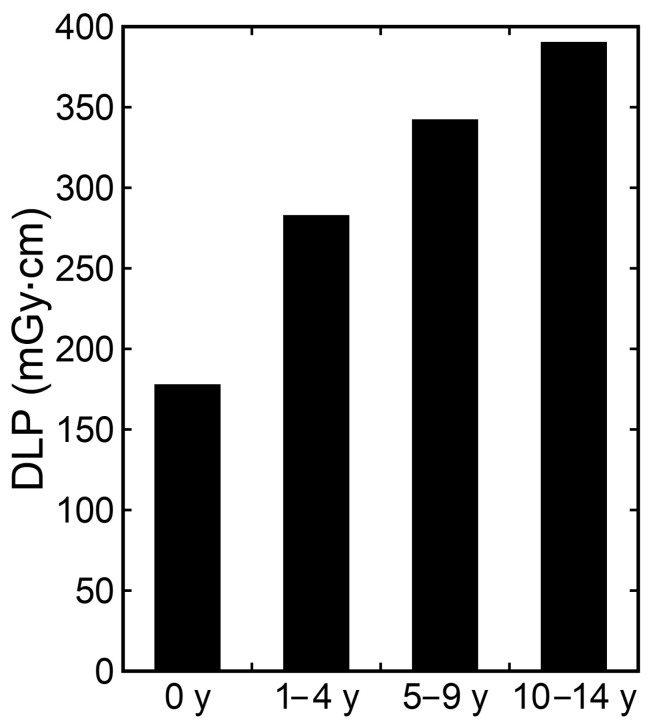
Median DLP for each age group.

**Figure 6:**
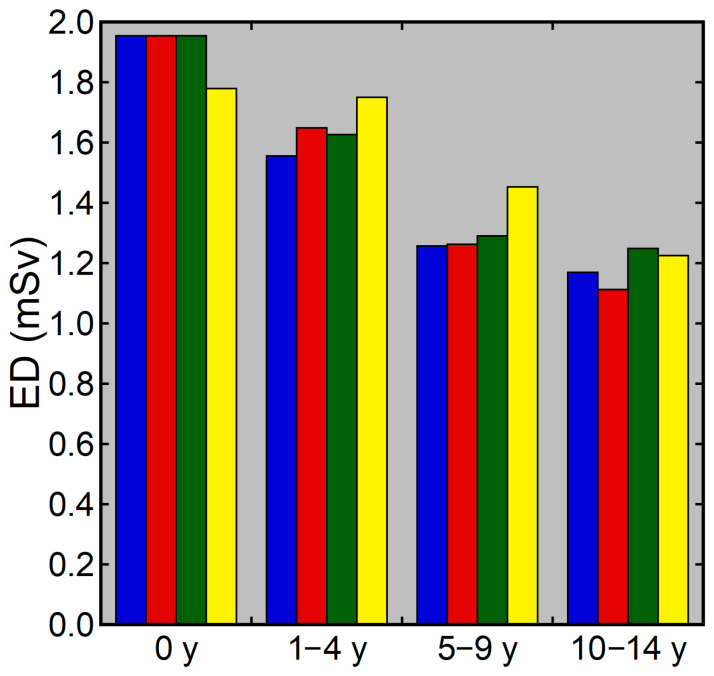
Median ED for each age group. The blue, red, green, and yellow bars represent EDs estimated by the curve method, linear method, simple method, and Radimetrics method, respectively.

In addition to analysis for each age, analysis was performed for the 0-, 1–4-, 5–9-, and 10–14-year age groups. The median values of the DLP and the EDs estimated by the four methods for each age group are presented in Figure 5 and Figure 6, respectively. The median DLP was higher and the median EDs were lower in the older groups than in the younger groups. The median ED estimated by the curve method differed to a large extent between the 0- and 1–4-year groups and between the 1–4- and 5–9-year groups, and to a small extent between the 5–9- and 10–14-year groups. The median EDs estimated by the Radimetrics method differed from those estimated by the other three methods. The differences between the curve and linear methods and between the curve and simple methods tended to be smaller.

**Figure 7:**
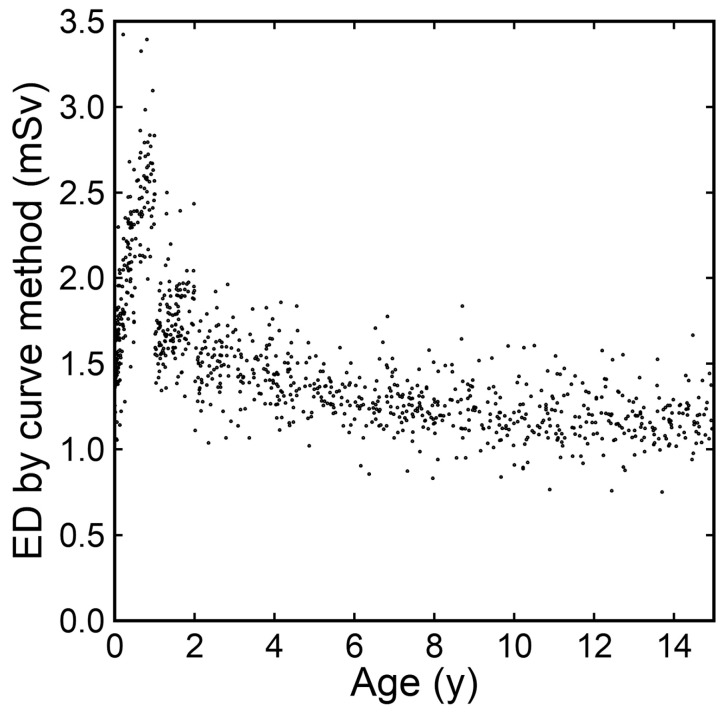
EDs estimated by the curve method plotted against age in actual number of years.

When the ED estimated by the curve method for each scan was plotted against age in actual number of years, the ED was relatively low immediately after birth and increased up to the age of 1 year (Figure 7). After that, the ED decreased and then plateaued.

**Figure 8:**
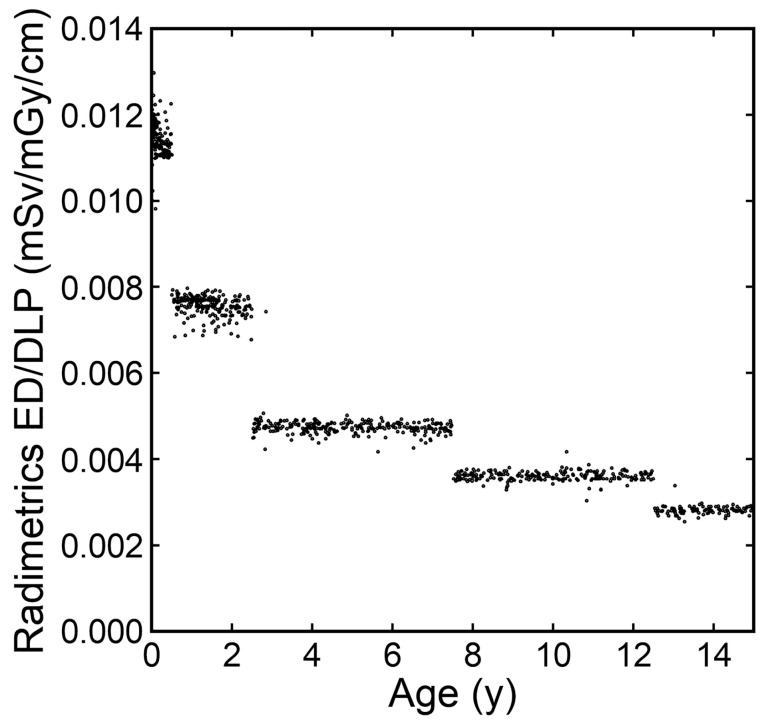
The ratios of the ED estimated by the Radimetrics method to the DLP plotted against age in actual number years.

The ratio of the ED estimated by the Radimetrics method to the DLP was plotted against age in actual number of years (Figure 8). The plots were visually classified into age groups of 0–0.5, 0.5–2.5, 2.5–7.5, 7.5–12.5, and 12.5–15 years. The mean (SD) ratios were 0.0115 (0.0004), 0.0076 (0.0002), 0.0047 (0.0002), 0.0036 (0.0001), and 0.0028 (0.0001) mSv/mGy/cm at 0–0.5, 0.5–2.5, 2.5–7.5, 7.5–12.5, and 12.5–15 years, respectively. The variations in each group were small, with a maximum coefficient of variation of 4.4% for the 2.5–7.5-year group.

## 4. Discussion

The ED represents the risk of stochastic effects of radiation and aids in comparing radiation doses from different radiation sources and for different diagnostic technologies. The ED in CT is often estimated by multiplying the DLP by a conversion factor. This method is convenient; however, the ICRP only provides conversion factors for children aged 0, 1, 5, and 10 years, and determination of an appropriate conversion factor is a concern when estimating the ED at other ages. In this study, we determined the conversion factor at each age by three methods (curve, linear, and simple methods) and estimated the ED.

The conversion factor provided by the ICRP decreases with increasing age. In the curve method, we simulated age-dependent changes in conversion factors using a single function to calculate a conversion factor for each age. The equation form y = a(x + b)^c^, selected empirically but not theoretically, achieved successful fitting, showing agreement with the ICRP values at 0, 1, 5, and 10 years and reasonable, consistent changes with age. The conversion factor calculated at each age was used to estimate the ED by the curve method. 

The median DLP at each age was lowest at 0 years and increased with age, reflecting head growth and a corresponding increase in tube current modulated by AEC. In contrast, the median ED estimated by the curve method was highest at 0 years and decreased with age. The annual decrease was gradually reduced, and the median ED plateaued at 9 years. It is indicated that the age-dependent decrease in the conversion factor initially exceeded and then counterbalanced the age-dependent increase in the DLP. It should be noted that the ED may be higher in younger children, even with a lower DLP. Furthermore, the risk of cancer induction is higher in younger children, even at the same ED. Justification and optimization are especially important in young children.

Unlike the curve method, the conversion factor for estimating the ED at each age may be selected from the four conversion factors provided by the ICRP, as in the simple method in this study. We used the value provided for the closest age, and the ICRP conversion factors at 0, 1, 5, and 10 years were applied to estimate the ED by the simple method in children aged 0, 1–2, 3–7, and 8–14 years, respectively. Actually, an appropriate conversion factor should decrease each year. The simple method is expected to overestimate the ED at 2 years because the conversion factor for a 1-year-old is applied to a 2-year-old child, and to underestimate the ED at 3 years because the conversion factor for a 5-year-old is applied to a 3-year-old child. The median ED estimated by the simple method showed complicated, unreasonable changes with age, suggesting limitations of the method.

The conversion factor may be calculated by linear interpolation of those provided by the ICRP, as in the linear method in this study. The provided value shows a rapid initial decrease and a subsequent slow decrease with age. It can be inferred that the appropriate conversion factor should decrease more between 1 and 2 years than between 4 and 5 years; however, linear interpolation neglects such a change in the rate of decrease. In addition, determination of the conversion factor for children older than 10 years is problematic. We used linear extrapolation of values at 5 and 10 years, which is expected to overestimate the annual decrease in the conversion factor. Minor discrepancies between the curve and linear methods were found at 2–4 years and over 10 years. The age-dependent changes in the conversion factor and ED appeared more reasonable for the curve method than for the linear method, which intuitively suggests the superiority of the curve method to the linear method, although the lack of standards prevents scientific confirmation. 

The DRL is an important tool to optimize the radiation dose in radiological imaging. For pediatric brain CT, age grouping is recommended to establish DRLs [11,12]. Each facility determines median values of CTDIvol and DLP recorded in clinical practice for each age group, and the 75th percentile values of the medians reported from many facilities are defined as the DRLs. In this study, we grouped patients into 0-, 1–4-, 5–9-, and 10–14-year age groups [3,4] and determined the median DLP and ED for each age group. The median DLP values were lower than the DRL values in our country [20]. In line with the previous papers, the DLP was higher in the older groups than in the younger groups [3,15,19,20], and the ED was lower in the older groups than in the younger groups, irrespective of the estimation method [27,28]. The discrepancy in the median ED for each age group was small between the curve and simple methods. For the simple method, the median ED for the 1–4-year group was determined using the EDs at 1 and 2 years calculated with the 1-year conversion factor and those at 3 and 4 years calculated with the 5-year conversion factor. Overestimation of the ED at 2 years and underestimation at 3 and 4 years are expected. The counterbalance between the overestimation at 2 years and the underestimation at 3 and 4 years is considered to have reduced the discrepancy in median values for the 1–4-year group between the curve and simple methods. When estimating median values in age groups, the simple method may be sufficient, depending on the group definition. 

The ED estimated by the curve method for each scan was plotted against the age calculated considering years, months, and days after birth and expressed in actual numbers. The ED was low immediately after birth and increased up to 1 year after birth. The DLP in brain CT increases rapidly during the first year after birth due to head growth [21]. The ED estimates increased proportionally to the DLP because the same conversion factor was applied during the first year. Actually, the appropriate conversion factor may decrease during the first year. A conversion factor at any age in actual numbers can be calculated based on the fitting curve; however, changes in the calculated conversion factor during the first year should be large, and its validity is not guaranteed, resulting in a large degree of uncertainty. We think that the curve method should not be applied to the assessment of changes in the ED during the first year after birth.

Radimetrics, a radiation dose management system, estimates the ED using Monte Carlo simulation [26]. The median ED at each age in integer years estimated by the Radimetrics method showed up-and-down changes with age, unlike the smooth changes observed with the curve method. The ratio of the ED estimated by the Radimetrics method to the DLP differed among the 0–0.5-, 0.5–2.5-, 2.5–7.5-, 7.5–12.5-, and 12.5–15-year groups and was almost constant within each age group. The grouping corresponded to that for the automatic selection of a phantom used in the Monte Carlo simulation. The ratio of the ED to the DLP was almost constant when using a given phantom, indicating that the Radimetrics method is essentially equivalent to multiplying the DLP by a conversion factor for each age group. It appears that the use of Radimetrics has no advantage over a conversion-factor-based method to estimate the ED in pediatric brain CT.

The curve method does not require dedicated software or substantial addition of labor and demonstrates reasonable age-dependent changes in the ED, indicating the usefulness of this method. We recommend the use of the curve method to estimate the ED in relation to age. The ICRP also provides the conversion factors for pediatric body CT at 0, 1, 5, and 10 years. Although the curve method was applied only to brain CT in this study, this method is also expected to aid in ED estimation in pediatric body CT.

The limitations of this study should be noted. Only one CT model with one imaging protocol was used in this study. We used AEC software installed on GE scanners to modulate the tube current and set the noise index—the main parameter representing the noise level of images reconstructed by filtered back-projection—at a fixed value in children, irrespective of age. Radiation dose modulation differs depending on the type and settings of the AEC software [29,30,31], which may influence the relationship between age and ED as well as the absolute value of the ED. The relationship between age and ED should be investigated for each radiation exposure protocol, and the curve method will contribute to such investigation. Moreover, we evaluated radiation dose but not image quality in this study. For optimization of radiological imaging, the radiation dose should be reduced while preserving image quality and clinical utility. Although the image quality was accepted in our routine clinical practice, we did not examine whether our protocol was optimal. It is not guaranteed that the observed age-dependent changes are applicable to a thoroughly optimized protocol. Additionally, the application of the curve method to ED estimation in pediatric body CT should be examined in the future.

## 5. Conclusions

In this study, we evaluated the ED in pediatric brain CT where radiation exposure was modulated using AEC. A conversion factor at each age was calculated based on a single equation determined using those at 0, 1, 5, and 10 years provided by the ICRP and was multiplied by the DLP to estimate the ED. This curve method demonstrated reasonable changes in the ED according to age. Although the DLP increased with increasing age, the ED was highest at 0 years and decreased with age. The proposed curve method is expected to contribute to estimating the ED in pediatric CT according to age.

## Data Availability

The data are available upon reasonable request from the corresponding author.
